# Microbiome and metabolic features of tissues and feces reveal diagnostic biomarkers for colorectal cancer

**DOI:** 10.3389/fmicb.2023.1034325

**Published:** 2023-01-13

**Authors:** Jiahui Feng, Zhizhong Gong, Zhangran Sun, Juan Li, Na Xu, Rick F. Thorne, Xu Dong Zhang, Xiaoying Liu, Gang Liu

**Affiliations:** ^1^School of Life Sciences, Anhui Medical University, Hefei, China; ^2^Henan International Joint Laboratory of Non-coding RNA and Metabolism in Cancer, Henan Provincial Key Laboratory of Long Non-coding RNA and Cancer Metabolism, Translational Research Institute of Henan Provincial People’s Hospital and People’s Hospital of Zhengzhou University, Zhengzhou, Henan, China; ^3^Department of Oncology, BinHu Hospital of Hefei, Hefei, China; ^4^School of Biomedical Sciences and Pharmacy, The University of Newcastle, Callaghan, NSW, Australia

**Keywords:** colorectal cancer, gut microbiome, metabolomics, biomarkers, tissue, feces

## Abstract

Microbiome and their metabolites are increasingly being recognized for their role in colorectal cancer (CRC) carcinogenesis. Towards revealing new CRC biomarkers, we compared 16S rRNA gene sequencing and liquid chromatography-mass spectrometry (LC–MS) metabolite analyses in 10 CRC (T_CRC_) and normal paired tissues (T_HC_) along with 10 matched fecal samples (F_CRC_) and 10 healthy controls (F_HC_). The highest microbial phyla abundance from T_HC_ and T_CRC_ were Firmicutes, while the dominant phyla from F_HC_ and F_CRC_ were Bacteroidetes, with 72 different microbial genera identified among four groups. No changes in Chao1 indices were detected between tissues or between fecal samples whereas non-metric multidimensional scaling (NMDS) analysis showed distinctive clusters among fecal samples but not tissues. LEfSe analyses indicated Caulobacterales and Brevundimonas were higher in T_HC_ than in T_CRC_, while Burkholderialese, Sutterellaceaed, Tannerellaceaea, and Bacteroidaceae were higher in F_HC_ than in F_CRC_. Microbial association networks indicated some genera had substantially different correlations. Tissue and fecal analyses indicated lipids and lipid-like molecules were the most abundant metabolites detected in fecal samples. Moreover, partial least squares discriminant analysis (PLS-DA) based on metabolic profiles showed distinct clusters for CRC and normal samples with a total of 102 differential metabolites between T_HC_ and T_CRC_ groups and 700 metabolites different between F_HC_ and F_CRC_ groups. However, only Myristic acid was detected amongst all four groups. Highly significant positive correlations were recorded between genus-level microbiome and metabolomics data in tissue and feces. And several metabolites were associated with paired microbes, suggesting a strong microbiota-metabolome coupling, indicating also that part of the CRC metabolomic signature was attributable to microbes. Suggesting utility as potential biomarkers, most such microbiome and metabolites showed directionally consistent changes in CRC patients. Nevertheless, further studies are needed to increase sample sizes towards verifying these findings.

## 1. Introduction

Colorectal cancer (CRC) is the third most common cancer and the fourth leading cause of cancer related deaths worldwide, and the second cause of cancer death in China (Tian et al., 2016; Zhang et al., 2017). Over the last 20 years, CRC has been rapidly increasing in the population under 50 years old with predictions of 550,000 new CRC cases in 2022 with an estimated 50,630 deaths (Xia et al., 2022; Zheng et al., 2022). Approximately 41% of all CRCs occur in the proximal colon, 22% in the distal colon and 28% in the rectum (Thanikachalam and Khan, 2019). The exact etiology of CRC remains unclear, but both genetics and environmental factors play important roles in its occurrence and development (Gao et al., 2017). Contributing lifestyle variables including age, tobacco and alcohol consumption, lack of physical activity, increased body weight and diet (Foulkes, 2008; Johnson et al., 2013; Thomas et al., 2019). Currently, surgery, chemotherapy and radiation comprise the major treatment strategies for CRC, with surgical resection being most effective treatment for localized disease, while chemotherapy is the best option for patients with lymph node metastases (Haraldsdottir et al., 2014).

The early diagnosis of patients with CRC is critical. The 5-year survival rate could be up to 90% if CRC patients were diagnosed in the early stage (Zhang et al., 2017). Despite improvements in imaging technologies, the accurate diagnosis of CRC still represents a clinical challenge (Liao et al., 2015; Notarnicola et al., 2018). Endoscopy is increasingly used for CRC screening; however, this invasive technique suffers from poor patient compliance, and there is still widespread reluctance in the population associated with the procedure (Simon, 2016; Ladabaum et al., 2020). Thus, other clinical examination techniques are still needed for the early detection of CRC (Phua et al., 2014; Macias et al., 2020). Recently, noninvasive monitoring tests, such as molecular biomarkers have been promoted as alternative non-invasive diagnostic tools for CRC diagnosis (Liu et al., 2013; Hong et al., 2016; Xu et al., 2016; Zhang S. et al., 2020). Among these studies, cancer progression has been associated with changes in the microbiome and metabolomics of feces, plasma, serum and tissues, proposing these as potential new biomarkers for the screening of various cancers including CRC (Spratlin et al., 2009; Mamas et al., 2011; Zhang S. et al., 2020). Moreover, evidence has emerged that the changes in tissue and gut microbiome are not passive aftereffects of carcinogenesis but rather, play a mechanistic role linking various risk factors to CRC pathogenesis. Microbiome and metabolomics biomarkers have been considered important approaches to discover the potential biomarkers for monitoring CRC progression (Zhang et al., 2017). Notably, many of the known cancer risk factors are also key determinants of the structures and functions of microbiome (Yazici et al., 2017; Hale et al., 2018; Kim et al., 2020). For example, comparison of CRC patients with healthy control individuals showed distinct clusters and alterations in the composition of enteric archaea during tumorigenesis whereas CRC-associated fecal samples show significant enrichment and depletion of halophilic and methanogenic archaea, respectively (Coker et al., 2020). Furthermore, meta-analysis showed higher species richness in CRC-associated samples compared to controls, with the further discovery that specific microbiome such as *Fusobacterium Parabacteroides*, *Streptococcus*, and *Lachnospiraceae* are associated with CRC (Thomas et al., 2019). Intriguingly, gut enrichments of microbiome in CRC patients showed a rapid decline occurring in the early postoperative period, suggesting that they may serve as potential CRC biomarkers (Xie et al., 2017).

Other studies have shown that microbiome interact with their hosts mainly through signals triggered by microbial metabolites, and changes in microbial metabolic functions are implicated in CRC pathogenesis (Tian et al., 2016; Yang et al., 2019; Kim et al., 2020; Zhang Y. et al., 2020). Microbial metabolites are strongly associated with cancer progression, influencing host metabolism, cellular signal transduction, and immune responses (Davis et al., 2012; Buas et al., 2017). So rather than the microbiome themselves, their metabolome directly affects CRC development and pathogenesis (Puchades-Carrasco and Pineda-Lucena, 2017; Kim et al., 2020). Notably, microbial metabolites have been shown to occur differentially in the serum, plasma, and urine in CRC patients vs. control subjects (Tokunaga et al., 2018). Thus far, metabolomics-based methods have identified different substances associated with the degree of cancer progression (Buas et al., 2017; Tokunaga et al., 2018). In the literature, bioactive lipids, fatty acids, polyunsaturated fatty acids, secondary bile acids, and sphingolipids showed consistent alterations in CRC patients, suggesting that these may represent early events in carcinogenesis (Kim et al., 2020; Li et al., 2022). Other studies have shown significant overall associations between gut microbiome with metabolome and the incidence of CRC (Thomas et al., 2019; Kim et al., 2020). Hence, microbiome and metabolomic analyses of cancer tissues and feces are important microenvironment, being different from healthy people (Feng et al., 2015; Williams et al., 2015; Tian et al., 2016; Kim et al., 2020; Taddese et al., 2020).

The microbiome and metabolome of tissue and gut appear to be closely linked to the overall physiopathological status of an individual, and moreover found to be strongly associated with the degree of CRC progression and development (Kim et al., 2020; Li et al., 2022). Indeed, significant changes in microbiome and metabolites have also been reported in the cancer tissues or fecal samples of gastric and esophageal cancer (Xu et al., 2016; Tokunaga et al., 2018). However, less attention has been paid to the conjoint analysis of microbiome and metabolome changes and their combined association in cancer tissues and fecal samples in CRC. Thus, to better understand and validate the potential links, it is necessary to investigate tissue and gut microbiome and metabolome simultaneously in CRC patients. Herein, we profiled the microbial communities of cancer tissues and feces in CRC patients and healthy controls to identify biomarker microbiome using high-throughput sequencing of 16S rRNA. Then we used the same samples to undertake liquid chromatography-mass spectrometry (LC–MS) analyses of their metabolomic features. In addition, microbial genera and metabolite data of CRC were utilized for correlation analyses. In short, we present fecal and tissue microbiota signatures that are characteristics of CRC and their associations with their metabolomic and CRC pathogenesis. This approach aimed to define the dual microbial and metabolomic characteristics of cancer tissues and fecal samples associated with CRC, and to explore the potential biomarkers for diagnosis and prognosis of CRC. Our findings provide evidence and suggest a potentially practical direction for further targeted experiments and developing new CRC prevention strategies.

## 2. Materials and methods

### 2.1. Ethics statement

This study was approved by the institutional review board of Anhui Medical University (20200491).

### 2.2. Patients, sample collection, and group designations

Ten patients and ten healthy volunteers were recruited from the First Affiliated Hospital of Anhui Medical University, China from 2020 to January 2022 (Supplementary Table S1). Inclusion criteria were as follows: (1) all participants were older than 18 and younger than 61 at the time of sample collection. (2) Diagnosis of CRC was defined according to clinical, radiological, endoscopic and histological criteria, and without other disease, and the TNM classification system was used for staging of patients with CRC as having TNM stages II/III disease. (3) None of the patients or healthy volunteers were treated with antibiotics, colon-cleansing products, or hormones within 1 month, nor did they receive radiation or chemotherapy before sample collection.

Ten tissue and fecal samples were collected from ten CRC patients. Additionally, ten tissue and fecal samples, respectively, were collected from the ten healthy volunteers. All tissues were frozen in liquid nitrogen immediately after the operation and stored longer term at −80°C until extraction of total DNA and protein. Stool samples were collected in sterile centrifuge tube on ice, and then immediately transferred to the laboratory and frozen at −80°C for further analysis. CRC tissue samples and their paired normal tissues were designated as T_CRC_ (*n* = 10, sample No. T_CRC_1-10) and T_HC_ (*n* = 10, sample No. T_HC_1-10), respectively. Healthy fecal samples and CRC fecal samples were designated as F_HC_ (*n* = 10, sample No. F_HC_1-10) and F_CRC_ (*n* = 10, sample No. F_CRC_1-10), respectively.

### 2.3. Microbiota sequencing and LC–MS analysis

Microbiota sequencing and LC–MS analysis followed the scheme in Supplementary Figure S1. Microbial DNA was isolated from fecal samples using the MagPure Soil DNA LQ Kit (Magen, Guangdong, China) according to the manufacturer’s instructions. The extracted DNA was diluted to 1 ng/μl and used as the template for PCR amplification. The 343F/798R (343F: 5′-TAC GGR AGG CAG CAG-3′; 798R: 5′-AGG GTA TCT AAT CCT-3′) primers were used to amplify the 16S rRNA gene for fecal samples. For detailed PCR reaction methodology, readers are referred to Gao et al. (2017). The amplicons were purified with Agencourt AMPure XP beads (Beckman Coulter Co., United States) and quantified using Qubit dsDNA assay kit. Microbial DNA of cancer tissue, PCR amplification, cancer tissue microbiome sequencing library and sequencing of 16S rRNA genes were performed at LC-BIO Bio-tech, Ltd. (Hangzhou, China) using the Illumina Hiseq platform (PE250).

Tissue and fecal samples were both analyzed using LC–MS. Chromatographic separations were performed using a Thermo Scientific UltiMate 3000 HPLC system using an ACQUITY UPLC BEH C18 column (100 mm × 2.1 mm, 1.8 μM, Waters, United Kingdom) heated to 35°C for reverse phase separation. The column flow rate was 0.4 ml/min with the mobile phase consisting of solvent solutions A (water, 0.1% formic acid) and B (Acetonitrile, 0.1% formic acid). The injection volume was 4 μl for each sample with gradient elution conditions as follows: 0–0.5 min, 5% B; 0.5–7 min, 5 to 100% B; 7–8 min, 100% B; 8–8.1 min, 100 to 5% B; 8.1–10 min, 5% B. All the data matrixes were combined from both positive and negative ion data. The original LC–MS data were processed by Progenesis QI V2.3 (Nonlinear, Dynamics, Newcastle, United Kingdom) for baseline filtering, peak identification, integral, retention time correction, peak alignment, and normalization. The extracted data were then further processed by removing any peaks with missing values (ion intensity = 0) in more than 50% in groups, by replacing zero value by half of the minimum value, and by screening according to the qualitative results of the compound. Compounds with resulting scores below 36 (out of 60) points were also deemed to be inaccurate and removed. Tissue sample analyses were performed at LC-BIO Bio-tech, Ltd. (Hangzhou, China), and the fecal samples were analyzed by Oebiotech Biotech Co., Ltd. (Shanghai, China).

### 2.4. Bioinformatics, statistical analysis, and microbiome-metabolite association analysis

Microbial raw sequencing data of all 40 samples were received in FASTQ format. Poor quality (below an average quality score of 30) and short sequences (shorter than 200 bp) were removed using Trimmomatic software (version 0.35), and clean reads were clustered to generate Operational Taxonomic Units (OTUs) with a 97% similarity cutoff using Vsearch 2.4.2 (Rognes et al., 2016) after primer sequence removal. Representative OUT sequences were given a taxonomic assignment based on the SILVA microbial database using BLAST Version 2.60 (Release 111; Altschul et al., 1997). Fecal microbial communities of F_HC_ and F_CRC_ were analyzed using data from 20 fecal samples as described in Section 2.2, including 10 fecal samples from healthy volunteers and 10 from CRC patients (SRA accession number: SRR19633878, SRR19633895, SRR19633906, SRR19633874-SRR19633876, SRR19633880-SRR19633885, SRR1933887-SRR19633890, and SRR19633913-SRR19633916). Chao1 index were calculated using Mothur, and non-metric multidimensional scaling (NMDS) by using R v3.4.1 (R Core Team, 2017). Linear Discriminant Analysis Effect Size (LEfSe) was carried out between groups to determine the differentially abundant taxonomic features by using the non-parametric Kruskal-Wallis rank sum test. Venn diagram and heatmap analyses were performed by using the online cloud tools.^1^ Functional prediction analyses were performed by using Phylogenetic Investigation of Communities by Reconstruction of Unobserved States (PICRUSt) based on the 16S rRNA OTU membership (Langille et al., 2013). Correlation within the microbial taxonomic abundance was measured using Brownian distance covariance (Szekely and Rizzo, 2009), which is available *via* the online platform (see footnote 1).

All metabolite concentration information was exported to Excel and normalized by weight across all parallel samples before inclusion in the bioinformatics analysis. Different microbiome and metabolites were analyzed by using GraphPad Prism v7.0 and SPSS 22.0. Partial least squares discriminant analysis (PLS-DA) was performed by using R v3.4.1 (R Core Team, 2017) to analyze the clustering of individuals between or among groups. All the data were presented as mean ± standard deviation (SD). Associations were determined by Spearman rank correlation. To determine the differences between groups, the independent-samples *t*-test and the Mann–Whitney U test were applied for normally and non-normally distributed data, respectively. *p* < 0.05 was considered statistically significant.

To assess the overall association between microbial composition and metabolomic profiles, we computed correlations of microbial genera with metabolites and individual metabolites using the data from the CRC and control groups. We first reduced the dimensionality of data using ordination techniques, then, we calculated correlations between the first principal coordinate of microbiome data and the first principal component of metabolome data. As the microbial data were sparser than the metabolomics data, and accordingly we applied a looser criterion to consider several microbial genera detected in at least 20%, rather than 80% of the samples. Spearman’s correlation coefficient and its significance were calculated and its 95% confidence interval describing the overall trend. Pairwise correlations, R scores, and *p* values for the microbial genera and metabolites were calculated using the online cloud tools in R package, and also at https://cloud.oebiotech.cn/task/ and http://www.lc-bio.com/. Features determined with significant correlations (*p* < 0.05) were plotted as heatmaps.

## 3. Results

### 3.1. Microbial composition, diversity analysis, taxonomic alterations, and association networks of microbiome

**Figure 1:**
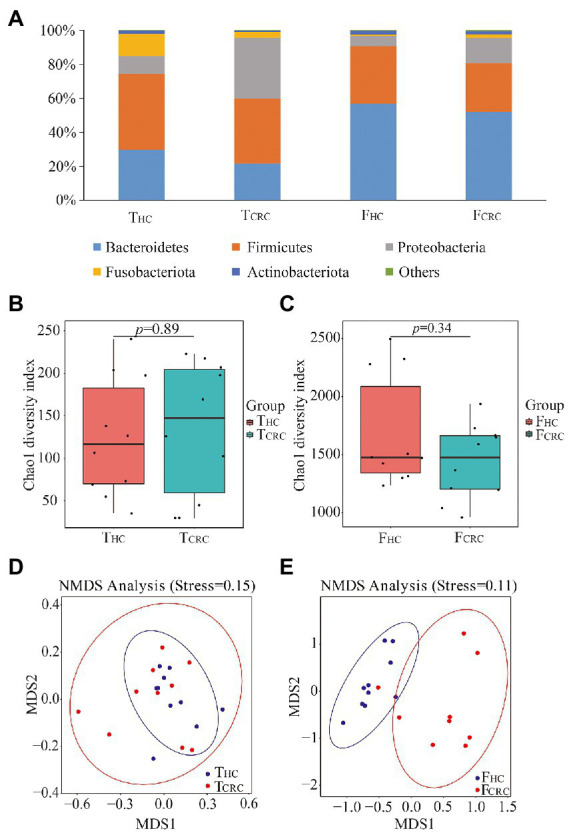
Microbial compositions at phyla level between T_HC_ and T_CRC_, and between F_HC_ and F_CRC_ **(A)** groups, respectively. Chao1 indices between the T_CRC_ and T_HC_ **(B)**, and between the F_CRC_ and F_HC_ groups **(C)**, respectively. NMDS analyses between the T_CRC_ and T_HC_ **(D)**, and between the F_CRC_ and F_HC_ groups **(E)**, respectively.

After sequencing the 16S rRNA genes of all 40 samples, data comparisons between the T_HC_ and T_CRC_ groups showed differences of 10 vs. 10 phyla, 21 vs. 19 classes, 30 vs. 36 orders, 60 vs. 66 families, and 222 vs. 309 genera, respectively. Comparisons between the F_HC_ and F_CRC_ groups showed differences of 21 vs. 33 phyla, 47 vs. 74 classes, 149 vs. 177 orders, 258 vs. 289 families, and 553 vs. 607 genera, respectively. The dominant microbial phyla from T_HC_ and T_CRC_ were Firmicutes, Bacteroidetes, Proteobacteria, Fusobacteria, and Actinobacteria, accounting for 41.27, 25.77, 23.12, 8.25, and 1.21% of the OTUs, respectively (Figure 1A). The dominant microbial phyla from F_HC_ and F_CRC_ were Bacteroidetes, Firmicutes, Proteobacteria, Proteobacteria, and Actinobacteriota, accounting for 54.46, 31.07, 10.50, 1.97 and 1.39% of the OTUs, respectively (Figure 1A). The highest abundance microbial phyla from T_HC_ and T_CRC_ were Firmicutes, while the dominant microbial phyla from F_HC_ and F_CRC_ were Bacteroidetes. Cancer tissue samples contained 32.94% unique microbial genera, while the fecal samples displayed 56.80% unique microbial genera with a total of 10.26% microbial genera in common between the cancer tissues and fecal samples.

To determine potential shifts in microbial communities of cancer tissues and feces between CRC and control samples, the alpha and beta diversities were analyzed. However, no changes in Chao 1 indices were detected between T_HC_ and T_CRC_, which was similar to the comparisons between F_HC_ and F_CRC_ (Figures 1B,C). Nonetheless, beta diversity comparisons between the samples using NMDS showed that all T_HC_ and T_CRC_ samples failed to cluster together (Figure 1D), whereas the F_HC_ and F_CRC_ samples clustered together (Figure 1E).

The raw data were analyzed to determine which microbiome were significantly associated with CRC patients compared with healthy volunteers. No significant differences were found between T_HC_ and T_CRC_ at the phyla or class levels. However, at the order level, Caulobacterales was found to be significantly less abundant in T_CRC_ compared with T_HC_. Moreover, family level differences of Corynebacteriaceae, Caulobacteraceae, and Veillonellaceae were dramatically different between T_HC_ and T_CRC_, and *Corynebacterium*, *Brevundimonas*, *Anaerovorax*, and *Acinetobacter* were significantly different at the genus level. Comparison of F_HC_ and F_CRC_ at the phyla level showed no substantial differences, although at the class level, Longimicrobia, Myxococcia, Brevinematia, Desulfuromonadia, and Alphaproteobacteria were significantly different. A total of 19 orders, 48 families, and 89 genera were identified between F_HC_ and F_CRC_ samples. A total of 72 different microbial genera were identified among T_HC_, T_CRC_, F_HC_ and F_CRC_ samples.

**Figure 2:**
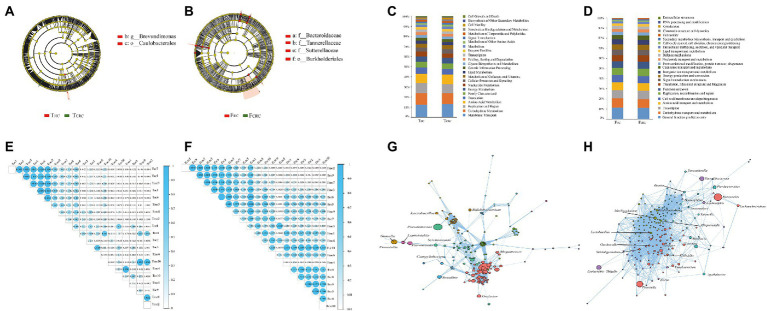
Most differentially abundant taxa between the T_HC_ and T_CRC_ **(A)**, and between the F_CRC_ and F_HC_ groups **(B)** based on LEfSe analyses, respectively. Functional predictions between the T_HC_ and T_CRC_ **(C)**, and between the F_CRC_ and F_HC_ groups **(D)** based on PICRUSt analyses, respectively. Correlation analysis in all samples of T_HC_ and T_CRC_ **(E)**, and the F_HC_ and F_CRC_ **(F)**, and the network analysis in T_HC_ and T_CRC_ **(G)**, and the F_HC_ and F_CRC_ **(H)**.

LEfSe was next used to determine the taxa that most likely reveal differences between CRC patients and control samples. Comparing T_HC_ with T_CRC_ samples showed that Caulobacterales and Brevundimonas were higher in T_HC_ (Figure 2A). Alternatively, comparing F_HC_ with F_CRC_ showed that Burkholderialese, Sutterellaceae, Tannerellaceaea and Bacteroidaceae were increased in F_HC_ (Figure 2B). Overall, 24 KEGG orthologs were identified between T_HC_ and T_CRC_, and between F_HC_ and F_CRC_, respectively. PICRUSt analysis results indicated that membrane transport (12.78%), carbohydrate metabolism (11.01%), replication and repair (9.60%), amino acid metabolism (9.27%) and translation (5.83%) were dominant functional predictions from comparisons between T_HC_ and T_CRC_ (Figure 2C). However, carbohydrate transport and metabolism (11.30%), transcription (8.82%), amino acid transport and metabolism (8.18%), cell wall/membrane/envelope biogenesis (7.61%), and replication, recombination and repair (7.21%) were the main predictions from the point of general function between F_HC_ and F_CRC_ (Figure 2D). To investigate individual relationships, we analyzed correlations between T_HC_ and T_CRC_ samples, with results showing positive correlations between T_HC_5, T_CRC_7, T_HC_1, T_HC_6, T_CRC_5 and T_HC_7, T_HC_5, T_CRC_7, T_HC_1, T_HC_6, respectively (Figure 2E). Comparing F_HC_ with F_CRC_ samples revealed that most samples had positive correlations with each other (Figure 2F). Moreover, we observed significant co-exclusive relationships between T_HC_ and T_CRC_-depleted microbiome including *Selenomonas*, *Megamonas*, and *Campylobacter* (Figure 2G). We also found that the genera including *Alistipes*, *Gardnerella*, *Lactobacillus*, *Dialister*, and *Alloprevotella* had substantially different correlation coefficients between F_HC_ and F_CRC_ (Figure 2H).

### 3.2. Metabolomic profiling of tissue and fecal samples

**Figure 3:**
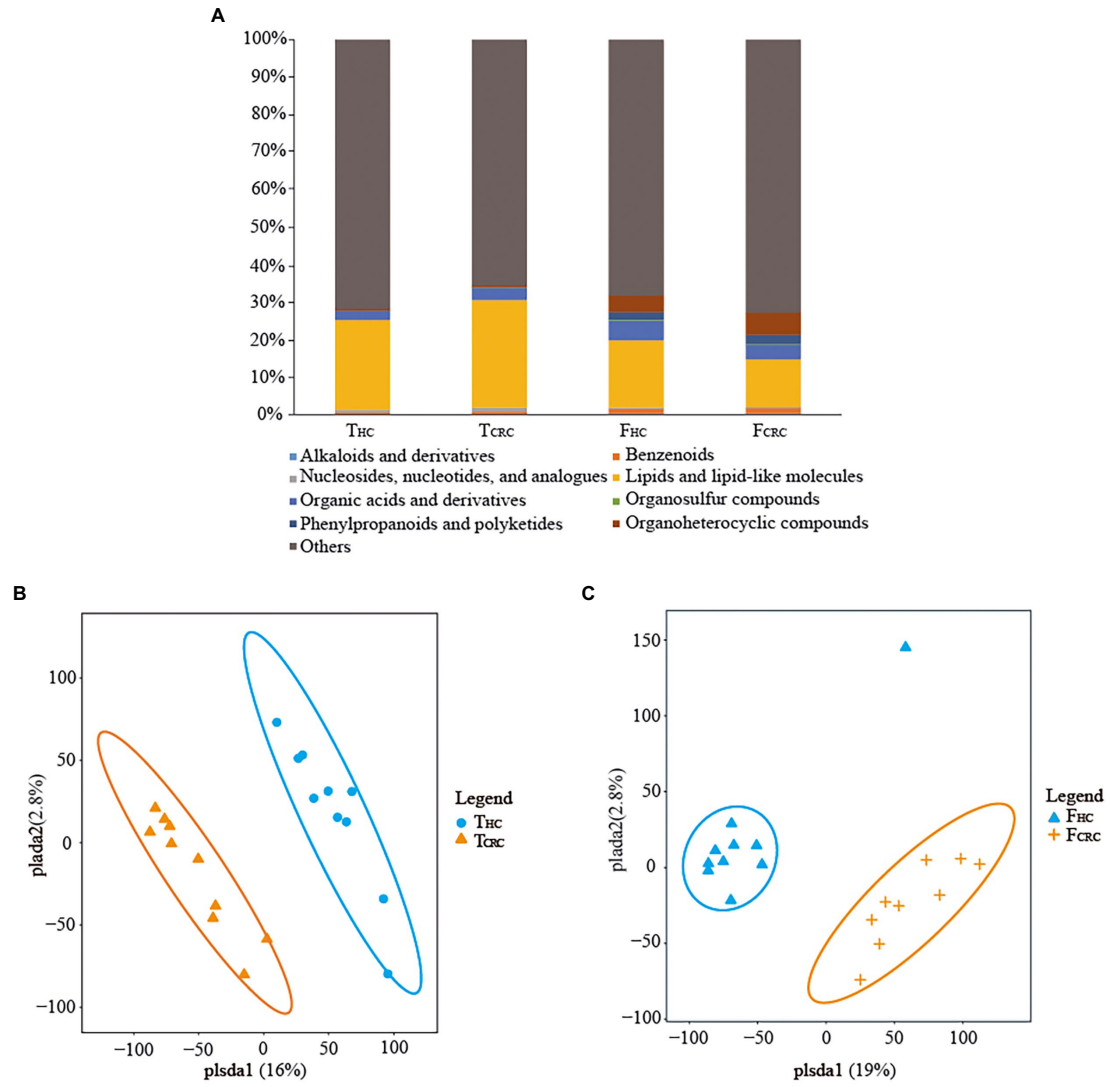
Compositions of metabolites between the T_HC_ and T_CRC_ groups, and between the F_HC_ and F_CRC_ groups **(A)**. PLS-DA score plots differentiating samples based on metabolites comparing the T_HC_ and T_CRC_ groups **(B)**, and between the F_HC_ and F_CRC_ groups **(C)**, respectively.

Metabolic analyses by LC–MS were successfully conducted on all matched cancer tissues and fecal samples, along with paired controls with the exception of one F_CRC_ sample. In general, over 854 metabolites belonging to 14 superclasses were detected in T_CRC_ and T_HC_, the dominant superclasses were lipids and lipid-like molecules, and organic acids and derivatives, accounting for 26.28 and 2.78%, the highest metabolite superclass includes lipids and lipid-like molecules, and the lowest being alkaloids and derivatives (Figure 3A). Notably, over 10,654 metabolites were identified in F_HC_ and F_CRC_ samples with the dominant abundant superclasses in rank order being lipids and lipid-like molecules and organoheterocyclic compounds, accounting for 14.90 and 7.09%, respectively (Figure 3A). Notably, like the tissue analyses, lipids and lipid-like molecules were the most abundant metabolites detected in fecal samples. Moreover, among the 854 and 10,654 metabolites identified, respectively, in tissues and feces, only 1.4% of metabolites were common to all four groups. Intriguingly, only 6.1% of metabolites were unique between T_HC_ and T_CRC_ samples in comparison to 92.5% unique metabolites between the F_HC_ vs. F_CRC_ samples. In particular, 10 differential metabolites were found among the four groups, namely N-lactoyl-Leucine, L-Kynurenine, Taurine, Myristic acid, 3Beta-7alpha-Dihydroxy-5-cholestenoate, 3-Formyl-6-hydroxyindole, 11-Hydroxyeicosatetraenoate glyceryl ester, Epitestosterone sulfate, Palmitelaidic acid, and 2,2-Dimethylsuccinic acid. To further illustrate the differences in metabolic profiles, the metabolomic data were subjected to PLS-DA analysis. Accordingly, we found all T_HC_ and T_CRC_ samples produced distinct clusters (Figure 3B) and similarly, all F_HC_ samples clustered distinctly from the F_CRC_ samples (Figure 3C).

### 3.3. Metabolic pathway analysis, altered metabolites analyses, and microbial-metabolite associations

**Figure 4:**
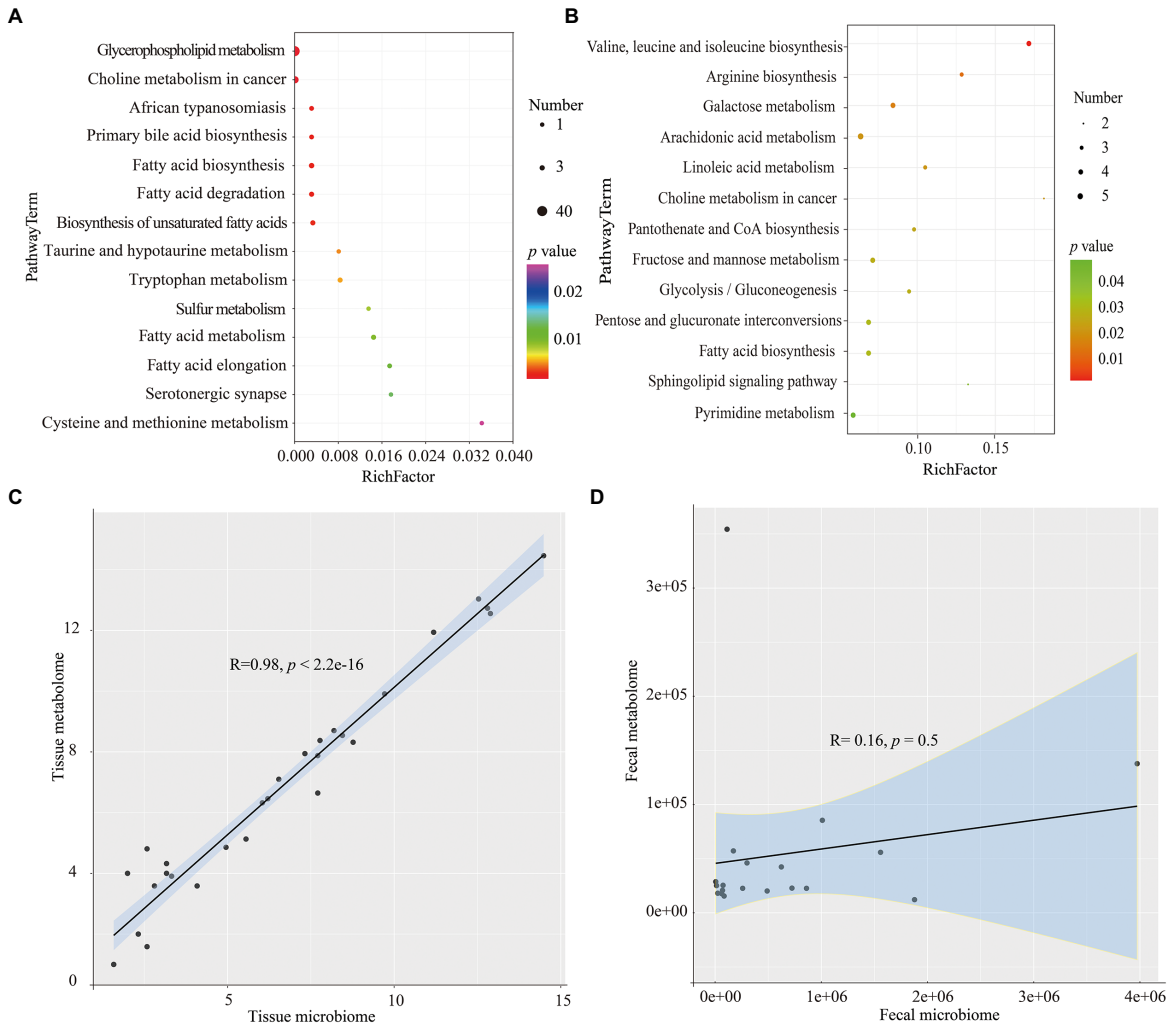
KEGG analyses of the differential metabolites the T_HC_ and T_CRC_ **(A)**, and between the F_HC_ and F_CRC_ groups **(B)**, respectively. Correlation between microbiome and metabolomics data for T_HC_ vs. T_CRC_ **(C)**, and F_HC_ vs. F_CRC_ **(D)**. *p < 0.05, **p < 0.01 and ***p < 0.001. Red: positive correlation; blue: negative correlation.

The differential metabolites identified above were next used to interrogate the KEGG compound database to identify related metabolic pathways. According to the findings, 14 pathways were found to be different between the T_HC_ and T_CRC_ groups, and among these, glycerophospholipid metabolism, choline metabolism in cancer and fatty acid biosynthesis were the most significantly different pathways (Figure 4A). Similar analyses comparing the F_HC_ and F_CRC_ groups uncovered 13 pathway enrichments, with the most prominent six pathways being arachidonic acid metabolism, valine, leucine and isoleucine biosynthesis, fructose and mannose metabolism, fatty acid biosynthesis, pyrimidine metabolism, and pentose and glucuronate interconversions (Figure 4B).

At the level of individual metabolites, 102 metabolites were differential between T_HC_ and T_CRC_ groups while 700 metabolites were differential between the F_HC_ and F_CRC_ groups. However, only one differential metabolite (Myristic acid) was detected amongst all four groups. In order to display the relationships among samples and metabolites more intuitively, we then analyzed the relationships among the samples using the top 50 significantly differential metabolites between T_HC_ and T_CRC_, and between F_HC_ and F_CRC_ groups, respectively. Except for a few individuals, the results showed that the samples in two groups can be distinguished with different metabolites occurring between the group comparisons (Supplementary Figures S2A,B).

We calculated the correlation between microbiome and metabolomics data for T_HC_ vs. T_CRC_, and F_HC_ vs. F_CRC_ comparisons. We found a highly significant positive correlation between microbiome and metabolomics data for T_HC_ vs. T_CRC_ (R = 0.98, *p* < 2.2 × 10^−16^), with similar results obtained between microbiome and metabolomics data for F_HC_ vs. F_CRC_; however, the R and *p* values were not significantly different (R = 0.16, *p* = 0.5; Figures 4C,D). To investigate the relationship of microbial taxa and metabolites, we analyzed the correlations between the top 20 abundance microbial genus profiles and the top 20 metabolites profiles. Comparing T_HC_ with T_CRC_ tissue samples showed positive correlations between the abundance of the *Prevotella* genera with L-Glutathione (reduced); *Parvimonas* and *Gemella* with Inosine; *Parvimonas* with L-Glutathione (reduced); *Dialister* with Erucamide. Conversely, the *Clostridium* genus was negatively correlated with LysoPE 18:0, LysoPC 16:0, 1-Oleoyl-sn-glycero-3-phosphocholine and LysoPC 18:0, respectively (Supplementary Figure S3A). Comparing F_HC_ and F_CRC_ fecal samples mainly showed that *Subdoligranulum* and *Prevotella* were positively correlated with P-Chlorophenylalaninee, Szopiclone, Eplerenone, and Porson, respectively. *Klebsiella* also showed positive correlations with THA, 6,9,12,15,18,21-Tetracosahexaenoic acid, mesobilirubinogen, respectively (Supplementary Figure S3B).

## 4. Discussion

The incidence of CRC is rising worldwide and while colonoscopy is an effective screening tool for CRC diagnosis, it remains unpopular with the subjects being tested. Hence, there is an unmet need to develop effective non-invasive examinations to detect the early development of CRC (Chen et al., 2015; Li et al., 2022). Microbiome and their metabolites are now known to play important roles in tumorigenesis with alterations in their composition and structures apparent in cancer tissues and feces (Zhang Y. et al., 2020). Therefore, a potential biomarker approach involves assessing the combination of stool microbiome and metabolites (Li et al., 2022). Towards the notion of providing non-invasive tests for CRC, this study profiled the microbiome and metabolites of cancer tissues and feces of CRC patients and compared these with samples from healthy volunteers using a combination of high-throughput sequencing and LC–MS technology. This analysis revealed promising data showing alterations in specific microbiome and metabolites in both CRC tissues and corresponding fecal samples. Like a previous report, we found that the gut microbiome in CRC tissues has greater richness than controls (Thomas et al., 2019), with common findings suggesting these studies collectively identify potentially useful microbial biomarkers for the design of non-invasive diagnostic tools to target CRC. It is known that early diagnosis and detailed staging of CRC significantly impact CRC management and outcomes (Zhang et al., 2017), with our study analysis advancing the diagnostic implications of microbial and metabolomic profiling as early detection approaches for CRC.

Pathogenic microbiome in CRC tissues or gut can influence the cellular microenvironment, leading to cancer development or otherwise promoting cancer progression. Changes in the balance of commensal microbiome may lead to a rise in mucosal permeability, microbial translocation, and activation of factors of the innate and adaptive immune system to stimulate chronic inflammation (Vacante et al., 2020). Indeed, gut microbiome have been emerged as one of central players in CRC pathogenesis, with multiple effects on the cancer transformation process and progression, and response to treatment of cancer (Helmink et al., 2019; Hasan et al., 2022). Microbiome are believed to contribute to CRC risk by producing toxins or exoenzymes, influencing the defense against pathogens, and deregulating immune homeostasis (Hasan et al., 2022). Thus, studying the microbiome of cancer tissues and feces may help understand the underlying mechanisms (Zmora et al., 2018). We found that the microbial communities of cancer tissues and feces from CRC patients were signatures inextricably linked to the presence of malignancy. Similar to the results of previous studies, our results indicated that the alpha and beta diversities of microbial species in cancer tissues did not show differences (Hasan et al., 2022). Moreover, our data showed lower evenness (α-diversity) and species richness in T_CRC_ than in T_HC_ samples, the microbial compositions between T_CRC_ and T_HC_ being similar to previous findings (He et al., 2021). The alpha diversity of fecal samples by Chao1 index in the F_HC_ group was higher than that in F_CRC_ group, but with no significant differences between F_HC_ and F_CRC,_ which was similar to previous findings (Thomas et al., 2019). However, beta diversity analysis showed dramatic differences between F_HC_ and F_CRC_ samples, supporting the conclusion of Yu (2018). Previous research has shown that the gut microbiome in CRC has a greater richness than controls, partially due to the presence of oral cavity-associated species rarely found in the healthy gut (Manichanh, 2006; Le Chatelier et al., 2013; Thomas et al., 2019). Microbial composition analysis showed Firmicutes and Tenericutes had the highest and lowest abundance, respectively, in T_CRC_ samples. Notably, these results differ from the previous reports, showing higher abundances of Proteobacteria and Fusobacteria, and lower abundances of Bacteroidetes, Actinobacteria, and Firmicutes in cancer tissues (Ringel et al., 2015; Kim et al., 2018; Shah et al., 2018; Wang et al., 2020). Nonetheless, microbial composition analysis of F_CRC_ indicated high abundance of Bacteroidetes, Firmicutes and Proteobacteria, similar to the study by Yu (2018). The results from the PICRUSt analyses showed that cell growth and death, biosynthesis of other secondary metabolites, and cell motility were the most central functions in the microbial communities of the cancer tissues. However, extracellular structures, RNA processing and modification, and cytoskeleton were the major functions in the gut microbial communities, which were different from the functions of the microbial communities of the cancer tissues. The likely reasons are the different abundances of microbiome in CRC tissues and feces (Ringel et al., 2015; Kim et al., 2018; Shah et al., 2018; Yu, 2018; Wang et al., 2020).

Cancer-specific microbiome have been detected in CRC mucosal and/or fecal samples and not in healthy controls (Vacante et al., 2020). Several CRC biomarker genera were identified as potential biomarkers in our study including *Solobacterium*, *Porphyromonas*, *Fusobacterium*, *Streptococcus*, *Gemella*, and *Bifidobacterium*, consistent with previous research (Thomas et al., 2019). Notably, the great abundance of *Fusobacteria* has been observed in CRC and the species has been associated with poor prognosis in CRC patients and development of chemoresistance (Yu, 2018). *Fusobacterium* has been associated with colorectal tumors and adenomas (Thomas et al., 2019) and moreover, it was reported that *F. nucleatum* increased cell growth, invasiveness, and capability to form xenografted CRC tumors (Yang and Yu, 2018). However, our application of PICRUSt to the 16S rRNA amplicon sequencing data to infer microbial metabolic functions indicated no common microbial metabolic functions among the four groups, likely indicating these data reflect the different microbial communities.

Metabolite alterations have been reported in a variety of cancers, representing potentially important biomarkers for diagnosis, treatment and prognosis (Erben et al., 2018; Yachida et al., 2019; Zhang S. et al., 2020; Hasan et al., 2022). LC/MS-based metabolite profiles in cancer tissues and feces of CRC patients were compared in PLS-DA analyses, with the overall changes in metabolites providing excellent discrimination in cancer vs. normal comparisons, suggesting these were good candidates for biomarkers in CRC. Further dissection of the altered metabolic profiles between T_HC_ and T_CRC_, and between F_HC_ and F_CRC_ revealed that 10 metabolites were dramatically different among the four groups. Among these, N-lactoyl-leucine was reported to be associated with human kidney cancer detection (Knott et al., 2018) while Taurine metabolism represents an important regulatory pathway in breast cancer, and potential diagnostic measure (Huang et al., 2016). Taurine is a non-essential amino acid and an end product of sulfur metabolism, being essential for cell growth in renal, neural, and cardiac cells, preventing cell death (Baliou et al., 2020). Myristic acid, as a rare fatty acid, is dramatically decreased in endometrial cancer (Troisi et al., 2018). Palmitelaidic acid is rare in nature, but has been widely produced by food industry, however, previous research has indicated that high palmitelaidic acid consumption may increase cancer risk (Li et al., 2017). L-kynurenine, 3beta-7alpha-dihydroxy-5-cholestenoate, 3-Formyl-6-hydroxyindole, 11-hydroxyeicosatetraenoate glyceryl ester, Epitestosterone sulfate, and 2,2-Dimethylsuccinic acid were dramatically different between CRC and normal people in the first report. Pathway enrichment analysis showed that fatty acid biosynthesis was altered in CRC patients compared with that of healthy people in both cancer tissue and fecal samples. Fatty acids for membrane synthesis are common features in metabolism and are altered in energy metabolism in cancer cells (Currie et al., 2013). Akin to previous findings, differences in energy metabolism were clearly observable between the CRC tissues and feces and their control samples, a result likely associated with the high aerobic glycolysis rates associated with increased glucose uptake and utilization along with increased lactate production (Tian et al., 2016). Interestingly, six common metabolic pathways were observed between tissue samples and fecal samples, and we suspect these pathways provide potentially important clues for the further development of biomarkers.

Through a multi-omics profiling approach, we were able to investigate the associations between the microbiome and metabolites in CRC patients compared with normal subjects from both cancer tissues and fecal samples. Some microbiome and metabolites followed either positive or negative correlations in CRC patients compared with normal subjects. In cancer tissue samples, *Clostridium* was found to increase in abundance and was significantly correlated with four metabolites (LysoPE 18:1, LysoPE 18:0, LysoPC 16:0, 1-Oleoyl-sn-glycero-3-phosphocholine, and LysoPC 18:0), which may increase malignant progression during CRC progression (Xie et al., 2017; Coker et al., 2022). Moreover, our association analysis revealed that the compositions, structures and relationships between microbiome and metabolites were significantly different between T_HC_ and T_CRC_. Comparing F_HC_ to F_CRC_, *Subdoligranulum* and *Prevotella* were positively correlated with porson, eplerenone, eszopiclone, and P-chlorophenylalaninee. Collectively, the results of the relationships between microbiome and metabolites suggest potential biomarkers in the development of CRC.

The diversity and subject-specificity of the human microbiome and metabolite are not yet fully uncovered, many of them with unknown functions. Technological advancements, especially large-scale shotgun metagenomics can overcome these limitations. Although interesting findings were gained from this study, some limitations must be acknowledged such as the small study size of 10 sample per group. This necessitates alternative larger cohorts on which to further validate our findings. However, as no single biomarker screen can be considered to provide definitive evidence, the findings here still make important and significant contributions to the field. Indeed, the relationships between microbial and metabolic biomarkers are complicated by the diet and physiology of the host, placing value in studies from diverse populations. In any event, the current findings provide evidence to further explore new non-invasive diagnostic tools for CRC. Furthermore, it is probably that the study could be further supplemented by incorporating additional omic analyses, such as proteogenomics, metagenomics, and proteomics, to expand the functional relationship between the microbiome, their metabolites and host functions.

In conclusion, we found significant alteration of metabolites between T_HC_ and T_CRC_, while the microbial profiles were not significantly different. Most such microbiome and metabolites showed directionally consistent changes in the CRC patients, suggesting that those changes may represent early events of carcinogenesis. Our study suggests that metabolic compositional and functional dissimilarities in CRC characterized by alterations in biodiversity and composition of microbiome. However, the gut microbiome and metabolites were both altered compared with those of normal subjects. Thus, our results identify several microbiome and metabolites that may act as potential biomarkers in CRC. However, before considering these for use in non-invasive diagnostic strategies for CRC, it will be necessary to increase the numbers of samples to validate these findings. Moreover, the functional contributions of the putative biomarker microbiome and metabolites need further exploration.

## Data availability statement

The datasets presented in this study can be found in online repositories. The names of the repository/repositories and accession number(s) can be found at: https://www.ncbi.nlm.nih.gov/genbank/, SRR21189098-SRR21289117.

## Author contributions

GL and XL designed the experiments of this manuscript. JF, ZG, ZS, JL, and NX conducted sample collection and data analysis. JF, ZG, GL, and XL wrote the manuscript. RT and XZ contributed to discussions and suggestions. All authors contributed to the article and approved the submitted version.

## Funding

This research was supported by the National Natural Science Foundation of China (Grant no. 81772908).

## Conflict of interest

The authors declare that the research was conducted in the absence of any commercial or financial relationships that could be construed as a potential conflict of interest.

## Publisher’s note

All claims expressed in this article are solely those of the authors and do not necessarily represent those of their affiliated organizations, or those of the publisher, the editors and the reviewers. Any product that may be evaluated in this article, or claim that may be made by its manufacturer, is not guaranteed or endorsed by the publisher.

## Footnotes

^1^ https://cloud.oebiotech.cn/task/
