# Supplementary material for: Microbiome and metabolic features of tissues and feces reveal diagnostic biomarkers for colorectal cancer
Source: Front Microbiol. 2023 Jan 13;14:1034325. doi: 10.3389/fmicb.2023.1034325 (PMC9880203; doi:10.3389/fmicb.2023.1034325)
Supplement: Supplementary file 1 [file Data_Sheet_1.docx]

**Supplementary table**

**Supplementary Table 1**. Clinical characteristics of the CRC patients and health volunteer (Mean ± SD)

|  | CRC (n = 10) | HC (n = 10) |
| --- | --- | --- |
| Age | 57.05±11.58 | 54.42±11.40 |
| Gender |  |  |
| Male | 4 | 5 |
| Female | 6 | 5 |
| TNM stage |  |  |
| II | 6 |  |
| III | 4 |  |
| BMI | 22.88 ±3.42 | 22.11 ±4.12 |
| Country | China | China |

**Supplementary Figures**

**
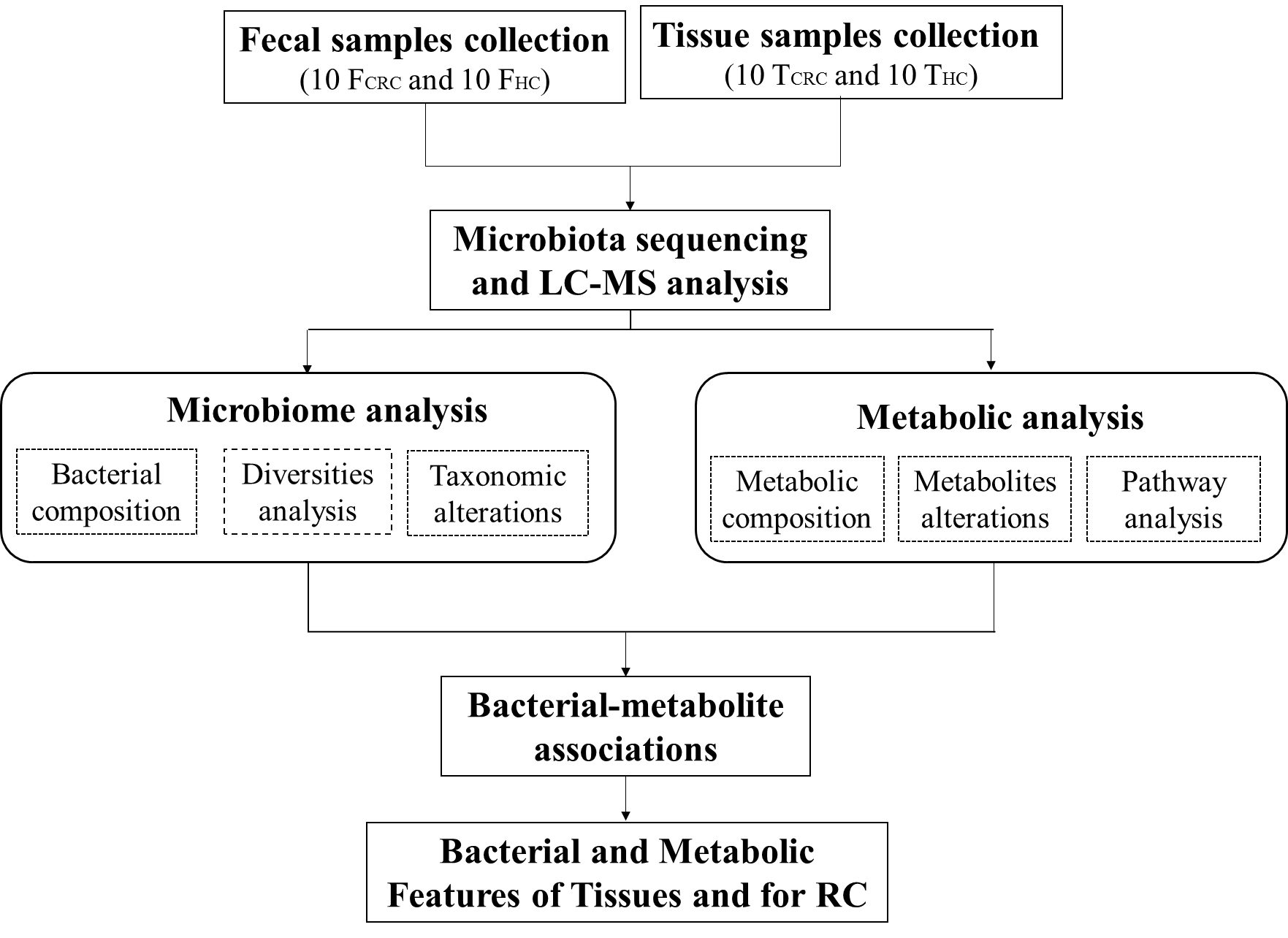
**

**Supplementary Figure 1.** Analysis flowchart of this study


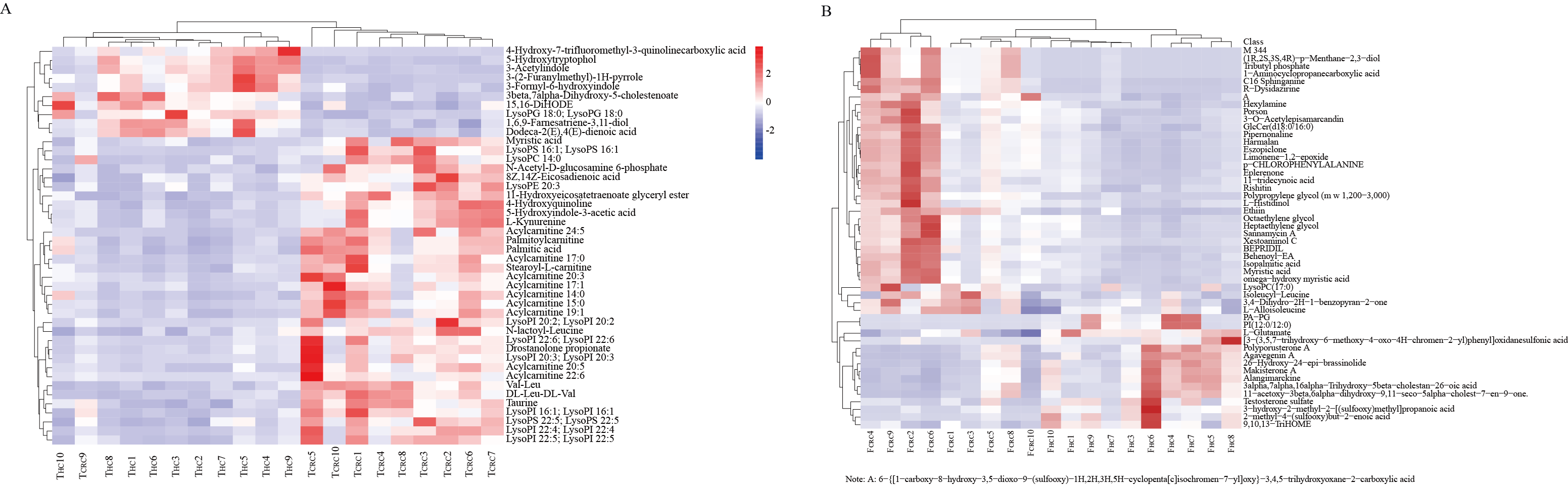


**Supplementary Figure 2.** Heatmaps of top 50 differential metabolites between the T_HC_ and T_CRC_ groups (A), and between the F_HC_ and F_CRC_ groups (B), respectively.


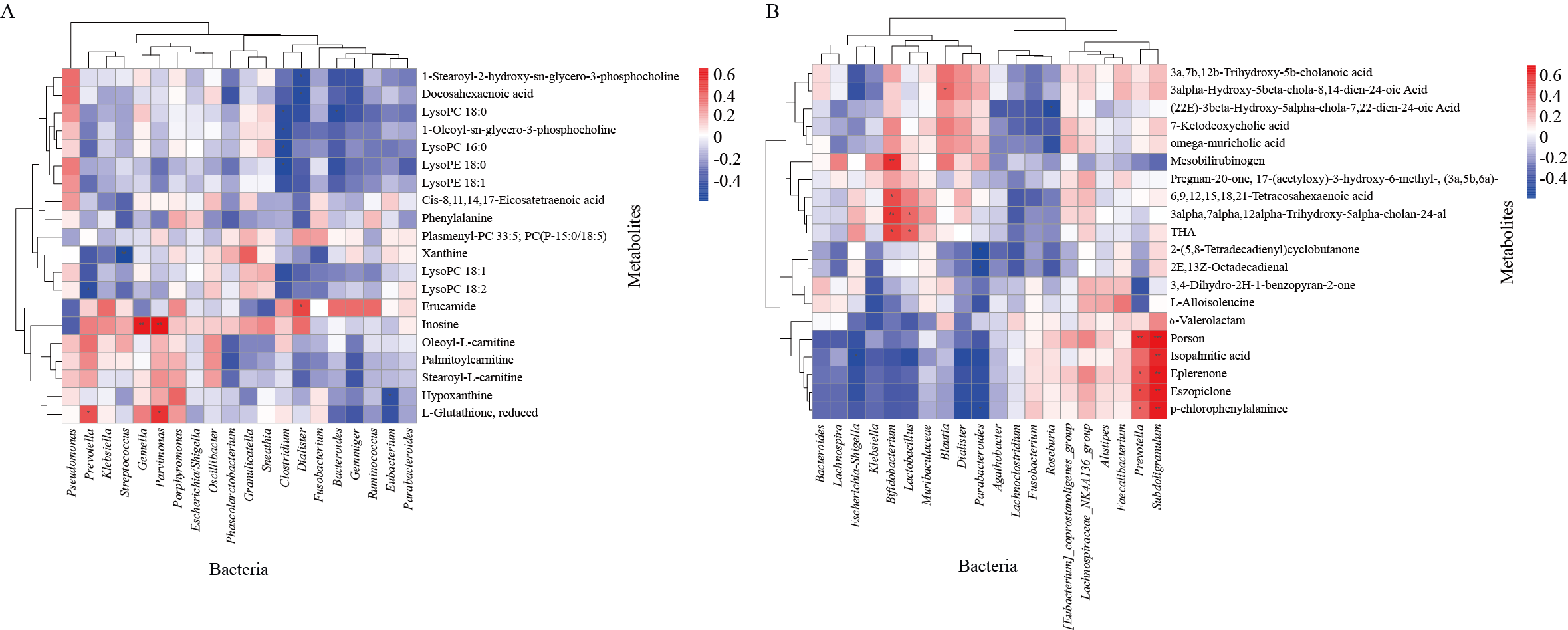


**Supplementary Figure 3**. Heatmaps illustrating correlations between the top 20 differential bacterial genera and top 20 differential metabolites in T_HC_ and T_CRC_ groups (A), and between the F_HC_ and F_CRC_ groups (B), respectively. Note: **p* < 0.05, ** *p* < 0.01 and *** *p* < 0.001. Red: positive correlation; blue: negative correlation.
